# Toward Identifying the Next Generation of Superfund and Hazardous Waste Site Contaminants

**DOI:** 10.1289/ehp.1002497

**Published:** 2010-10-01

**Authors:** Wendell P. Ela, David L. Sedlak, Morton A. Barlaz, Heather F. Henry, Derek C.G. Muir, Deborah L. Swackhamer, Eric J. Weber, Robert G. Arnold, P. Lee Ferguson, Jennifer A. Field, Edward T. Furlong, John P. Giesy, Rolf U. Halden, Tala Henry, Ronald A. Hites, Keri C. Hornbuckle, Philip H. Howard, Richard G. Luthy, Anita K. Meyer, A. Eduardo Sáez, Frederick S. vom Saal, Chris D. Vulpe, Mark R. Wiesner

**Affiliations:** 1 Chemical and Environmental Engineering, University of Arizona, Tucson, Arizona, USA; 2 Civil and Environmental Engineering, University of California–Berkeley, Berkeley, California, USA; 3 Civil, Construction, and Environmental Engineering, North Carolina State University, Raleigh, North Carolina, USA; 4 Superfund Research Program, National Institute of Environmental Health Sciences, Research Triangle Park, North Carolina, USA; 5 Aquatic Ecosystem Protection Research Division, Environment Canada, Burlington, Ontario, Canada; 6 Environmental Health Sciences Water Research Center, University of Minnesota, St. Paul, Minnesota, USA; 7 National Exposure Research Laboratory, U.S. Environmental Protection Agency, Athens, Georgia, USA; 8 Civil and Environmental Engineering, Duke University, Durham, North Carolina, USA; 9 Environmental and Molecular Toxicology, Oregon State University, Corvallis, Oregon, USA; 10 National Water Quality Laboratory, U.S. Geological Survey, Denver, Colorado, USA; 11 Veterinary and Biomedical Sciences, University of Saskatchewan, Saskatoon, Saskatchewan, Canada; 12 School of Sustainable Engineering and the Built Environment, Arizona State University, Tempe, Arizona; 13 Department of Environmental Health Sciences, Johns Hopkins University, Baltimore, Maryland, USA; 14 National Program Chemicals Division, U.S. Environmental Protection Agency, Washington, DC, USA; 15 Public and Environmental Affairs, Indiana University, Bloomington, Indiana, USA; 16 Civil and Environmental Engineering, University of Iowa, Iowa City, Iowa, USA; 17 Syracuse Research Corporation, Syracuse, New York, USA; 18 Civil and Environmental Engineering, Stanford University, Stanford, California, USA; 19 Environmental and Munitions Center of Expertise, U.S. Army Corps of Engineers, Omaha, Nebraska, USA; 20 Division of Biological Sciences, University of Missouri, Columbia, Missouri, USA; 21 Nutritional Science and Toxicology, University of California–Berkeley, Berkeley, California, USA

**Keywords:** contaminants of emerging concern, emerging contaminant, hazardous waste site, Superfund

## Abstract

**Background:**

This commentary evolved from a workshop sponsored by the National Institute of Environmental Health Sciences titled “Superfund Contaminants: The Next Generation” held in Tucson, Arizona, in August 2009. All the authors were workshop participants.

**Objectives:**

Our aim was to initiate a dynamic, adaptable process for identifying contaminants of emerging concern (CECs) that are likely to be found in future hazardous waste sites, and to identify the gaps in primary research that cause uncertainty in determining future hazardous waste site contaminants.

**Discussion:**

Superfund-relevant CECs can be characterized by specific attributes: They are persistent, bioaccumulative, toxic, occur in large quantities, and have localized accumulation with a likelihood of exposure. Although still under development and incompletely applied, methods to quantify these attributes can assist in winnowing down the list of candidates from the universe of potential CECs. Unfortunately, significant research gaps exist in detection and quantification, environmental fate and transport, health and risk assessment, and site exploration and remediation for CECs. Addressing these gaps is prerequisite to a preventive approach to generating and managing hazardous waste sites.

**Conclusions:**

A need exists for a carefully considered and orchestrated expansion of programmatic and research efforts to identify, evaluate, and manage CECs of hazardous waste site relevance, including developing an evolving list of priority CECs, intensifying the identification and monitoring of likely sites of present or future accumulation of CECs, and implementing efforts that focus on a holistic approach to prevention.

During the late 1970s, environmental toxicologists began to recognize that communities living adjacent to hazardous waste sites were sometimes exposed to toxic contaminants. This recognition motivated policy makers to develop regulations intended to reduce exposures and to prevent similar exposures from occurring in the future (Acton 1989; Hird 1994). Increased awareness of risk and the availability of new funding has motivated a new era of research on chemical fate, toxic effects, and environmental remediation. To prioritize monitoring and research efforts, environmentalists developed lists of contaminants that were based on specific factors that included production volume, toxicity, and the availability of suitable analytical techniques (Keith and Telliard 1979). Over the past 30 years, these lists have guided site cleanup efforts and fundamental research that have focused almost all attention on a relatively small number of contaminants that are frequently detected at hazardous waste sites such as polychlorinated biphenyls (PCBs), halogenated solvents, arsenic, and benzene. This focus has resulted in the development of regulations, policies, and remedial actions that have improved public and environmental health proximate to contaminated sites [U.S. Environmental Protection Agency (EPA) 2010a]. However, additional contaminants that were not included in the original lists likely pose risks to humans and biota (Birnbaum and Staskal 2004; Nolan et al. 2009; Richardson 2009). This article provides an overview of issues related to previously unrecognized contaminants relevant to hazardous waste sites and identifies research needs to address these particular contaminants of emerging concern (CECs).

Recent monitoring of municipal wastewater effluent, urban surface waters, and biota has documented the occurrence of groups of previously unrecognized contaminants. Much of the interest in these CECs can be traced to advances in analytical chemistry that have enabled the analysis of polar and thermally labile compounds by liquid chromatography coupled with mass spectrometry and tandem mass spectrometry (Kolpin et al. 2002; Schultz et al. 2004; Ternes 1998). Improvements in sample preconcentration (Mitch et al. 2003) and detection (Motzer 2001) also have contributed to the identification and quantification of novel CECs.

Because of their widespread use and environmental persistence, some CECs are ubiquitous and transported far from their sources where they may bioaccumulate. For example, polybrominated diphenyl ethers [PBDEs; e.g., decabromodiphenyl ether (DBDE), shown in Figure 1], which are flame retardants widely used in furniture and electronics, are known to accumulate in household dust and undergo long-range atmospheric transport (Hites 2004). In industrialized countries, PBDEs are routinely detected in human blood at concentrations of concern to environmental toxicologists (Birnbaum and Staskal 2004; Johnson et al. 2010). They also have been detected far from their sources in polar bears and gulls in the arctic (Verreault et al. 2005). Production and use of PBDEs have been restricted in the European Union and in some U.S. states. However, other brominated flame retardants with similar transport properties are now being used as replacements (Stapleton et al. 2008), and other halogenated flame retardants with similar properties continue to be produced in large quantities (Muir and Howard 2006). PBDEs and other hydrophobic CECs partition into organic phases (e.g., biosolids from municipal sewage treatment plants) and may consequently accumulate at land disposal sites (U.S. EPA 2009; National Research Council 2002).

## Contaminants of Concern at Hazardous Waste Sites

**Figure 1:**
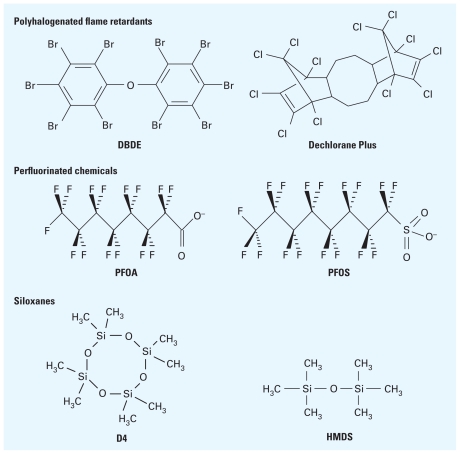
Some potential CECs relevant to the Superfund. Abbreviations: D4, octamethylcyclotetrasiloxane; DBDE, decabromodiphenyl ether; HMDS, hexamethyldisiloxane; PFOA, perfluorooctanoate; PFOS, perfluorooctane sulfonate.

Relatively little attention has been directed toward the occurrence of CECs at existing hazardous waste sites or to the possibility that the use or disposal of CECs may create new hazardous waste sites. Nevertheless, increased monitoring has identified cases in which the contamination of CECs has been linked to industrial use and to the disposal of chemicals (Phillips et al. 2010). For example, in a German study, Skutlarek et al. (2006) reported that land disposal of organic waste that contained perfluorinated compounds caused elevated concentrations of perfluorooctanoate (PFOA) and perfluorooctane sulfonate (PFOS) (Figure 1) in the Ruhr river that were considered harmful to human health. In another study, Hoh et al. (2006) noted that CECs that were discovered during routine monitoring were later traced back to industrial sites where the flame retardant Dechlorane Plus (Figure 1) had been released near Niagara Falls, New York, USA. In an earlier report, Motzer (2001) found that perchlorate had been introduced into the lower Colorado River near Las Vegas, Nevada, USA (Motzer 2001).

In some cases, CECs have been detected after remediation of priority contaminants, which has delayed or prevented site closure. For example, the hydrophilic compound 1,4-dioxane, commonly used as a stabilizer for 1,1,1-trichloroethane, may remain in groundwater after bioremediation or soil vapor extraction. Failure to initially recognize this compound’s presence in trichloroethane-contaminated groundwater necessitated additional remediation of several sites that were presumed to be clean (Zenker et al. 2003). Nitrosodimethylamine (NDMA), a compound for which the detection limit using standard analytical methods is relatively high, required additional remediation at sites when more sensitive analytical methods were employed (Mitch et al. 2003). Currently, no fixed screening list of chemicals has been developed for the U.S. EPA and the Department of Defense sites, and only a few hundred chemicals are screened during site characterization. Thus, screening is focused on a small list of contaminants or relies on less sensitive analytical methods than are currently available. These factors are counterproductive to remediation and, in some cases, may increase exposure to CECs.

The cases cited above suggest that research on the fate, effects, and remediation of contaminants at hazardous waste sites be expanded to include CECs. However, it is the opinion of the authors that simply expanding the priority list by including additional contaminants as they are discovered is an inefficient way to protect public health. In fact, developing a proactive strategy to address waste-site–relevant CECs requires a better understanding of the properties of chemicals that may cause the greatest threats to public health near waste sites.

For insight into the process of identifying candidate CECs, numerous scientists recently have attempted to prioritize chemicals classified as persistent, bioaccumulative, and toxic (Arnot and Mackay 2008; Brown and Wania 2008; Howard and Muir 2010; Mitchell et al. 2002). Because data on the physical and chemical properties of most chemicals in commerce are lacking, environmentalists generally prioritize candidate chemicals using quantitative structure–property relationships (QSPRs) to predict key environmental fate and distribution indicators such as octanol–water partitioning; air–water partitioning and bioconcentration; and rates of biotransformation, hydrolysis, atmospheric oxidation, and photolysis (Howard and Muir 2010). Data on production volume also can guide the prioritization process (Arnot and Mackay 2008; Howard and Muir 2010). Data on toxicity, measured or predicted from quantitative structure–activity relationships (QSARs), provide additional insight (Arnot and Mackay 2008).

Because of the limitations of QSPRs and QSARs, expert judgment is relied upon to identify data gaps and to further prioritize CEC research and monitoring. Although this reliance on subjective judgment often is at odds with efforts to remove potential bias, it is an effective way to bring new research findings into the prioritization process.

Similar approaches may be useful in identifying CECs relevant to hazardous waste sites, provided they differentiate between contaminants that are widely dispersed at low concentrations and those that are localized at field sites (Table 1). This is especially challenging for existing algorithms that prioritize chemicals on the basis of persistence, because conditions in groundwater and sediments at hazardous waste sites (e.g., absence of light and oxygen) can enhance CEC persistence that may lead to long-term exposure to compounds that might be classified as degradable by more general testing protocols. For example, NDMA persists for decades in groundwater but when exposed to sunlight, it undergoes photolysis and aerobic biotransformation on a time scale of just days (Mitch et al. 2003).

## Research Needs

The authors of this article met at a workshop in August 2009 that was sponsored by the National Institute of Environmental Health Sciences (NIEHS) to identify CECs relevant to the Superfund and to other hazardous waste programs (NIEHS 2009). The group discussed potential classes of CECs (Figure 1) and processes that may lead to future hazardous waste sites. The discussions also identified knowledge gaps that hinder our ability to identify, characterize, avoid, or remediate these CECs.

Research needs fall into four broad areas: detection and quantification, environmental fate and transport, health effects and risk assessment, and site investigation and remediation approaches (Table 2). The following descriptions of research gaps are illustrative, not exhaustive.

### Detection and quantification

#### Final disposition of CECs

The end-of-life disposition of CECs and CEC-containing products is mapped poorly, and screening at disposal sites is inadequate to assess potential risk. Particularly relevant sites include electronic waste (e-waste) recycling or disposal sites, municipal solid waste landfills, and biosolids disposal sites. For example, PBDE, Dechlorane Plus, and PCB levels are elevated at e-waste recycling sites (Luo et al. 2009; Ren et al. 2009; Yuan et al. 2008). Similarly, consumer products (food packaging, cookware, textiles, and carpet) that contain fluorochemicals are commonly disposed of in landfills. Fluorochemicals have been detected in landfill leachate, which is typically treated in wastewater treatment plants (Busch et al. 2010). Some fluorochemicals or their degradation products either are unattenuated during biological treatment or partition to biosolids that may be applied to the land (Higgins et al. 2005; Schultz et al. 2006).

#### Bioassay-directed methods

More effort is needed to measure potentially toxic contaminants in complex environmental matrices. Enhanced and expanded arrays of bioassay-directed methods and bulk chemical screening methods can be coupled with chemical identification, and when a positive response is observed, instrumental techniques can be used to identify the novel compounds (Furuichi et al. 2004). The bioassay-directed fractiontion and identification methodology used to identify CECs can be linked to a particular end point (Gracia et al. 2008), such as mortality, or to a specific biochemical effect, such as estrogenicity (Snyder et al. 2000). This approach may be facilitated by recent developments in microarrays (Poynton and Vulpe 2009).

### Environmental fate and transport

#### Transformation processes

Some CECs are transformed readily into compounds with toxicities, bioavailability, and environmental mobility substantially different than the parent compound. Often only a single or small number of the possible transformation products are evaluated. Thus, exposure and effects related to contaminant releases at waste sites may not be well correlated with the parent compound. For example, the behavior and estrogenic potency of nonylphenol ethoxylates depend on the length of the ethoxylate chain and presence of carboxylate moieties (Routledge and Sumpter 1996; Teske and Arnold 2008). Transformation by natural and engineered processes shortens the ethoxy chain, which alters the compound’s transport and toxicity (Montgomery-Brown and Reinhard 2003). There are many similar examples for other CECs (Kolpin et al. 2009).

#### Fate and transport models

Current fate and transport models are not applicable to several classes of the CECs. For example, the unique chemical properties of perfluorinated chemicals that promote their use as stain blockers in fabrics (i.e., the strong tendency for the fluorinated alkyl chains to aggregate) also prevent the use of typical physicochemical properties (e.g., octanol–water partition coefficient) to accurately predict their mobility in aquatic ecosystems. The properties of several other classes of CECs make it difficult to measure their physicochemical properties. For example, the accurate measurement of the molecular properties of the organosilicones (e.g., Henry’s law constant) is difficult because of their high volatility and hydrophobicity (David et al. 2000). Similarly, commonly used models for predicting environmental fate and transport are not adequately developed to address the unique properties of nanoparticles that may exhibit properties midway between particulate and dissolved contaminants.

### Health effects and risk assessment

#### Unconventional responses and impacts

Research gaps in Superfund-relevant CECs are often gaps for CECs and environmental contaminants in general. For example, toxicologists have identified a need to understand the risks posed by compounds that do not exhibit a monotonic dose–response curve or affect different physiological end points differently in various concentration ranges (Talsness et al. 2009). There is a need to understand the effect of mixtures when exposure occurs in complex media (Conroy et al. 2007; Rodman et al. 1991), to link *in vitro* experimental results to toxicological manifestations in the whole organism, and to assess effects on sensitive subpopulations such as infants from chronic exposure. Although these needs are not unique to waste-site–relevant CECs, our ability to respond to hazardous waste site generation is compromised without additional research in these areas.

#### Bioaccumulation models

Existing models do not accurately predict bioaccumulation of many CECs (e.g., highly polar, ionized, or functionalized molecules). Perhaps the best example is the biomagnification (Houde et al. 2006) of PFOA and PFOS, which is not predicted from their relatively low octanol–water partition coefficients. Several other contaminants such as perchlorate and DBDE have been detected in tissues and organisms, which also is unanticipated using standard models (Cheng et al. 2008; Yu et al. 2002). For molecules of the size and hydrophobicity of DBDE (molecular weight, 959 Da), bioaccumulation via passive diffusion through membranes is predicted to be low. Nevertheless, there is growing evidence of uptake of this and other brominated flame retardants of similar molecular weight, suggesting that other absorption routes may be important (Thomas et al. 2005). This deficiency is exacerbated by a lack of bioaccumulation studies and measured data on exposure to a wide range of polar, ionized, and high-molecular-weight chemicals in humans and wildlife food chains.

### Site investigation and remediation approaches

#### Epidemiologically and ecologically focused geospatial analyses

The degree of exposure to CECs and whether they are responsible for unexplained or unrecognized ecological or health effects at hazardous waste sites is largely unexplored. Epidemiologically and ecologically focused geospatial analyses are not regularly employed to identify potential effects of concentrated sources of CECs. For instance, when blood samples were collected from workers at an e-waste dismantling site in China, the serum levels of PBDEs and the frequency of micronucleated and binucleated cells were significantly higher than those from a similar unexposed cohort (Yuan et al. 2008).

#### Remediation technologies

Remedial technologies for addressing CEC contamination are largely unstudied. The potential impairment of remediation strategies for priority pollutants due to the presence of CEC cocontaminants is also unknown. For example, no effective remediation technologies have been identified for triclosan and triclocarban, which are antimicrobial compounds that are detected at parts-per-million concentrations in sediments and biosolids (Miller et al. 2008; Wilson et al. 2008). The presence of these antimicrobial cocontaminants could promote multiple drug resistance in pathogens, inhibit microbial degradation of priority pollutants, or serve as alternative electron donors to reductively dechlorinating microorganisms, thereby slowing the natural attenuation rate of priority pollutants (e.g., PCBs). In some cases, it may be difficult to assess the efficacy of remedial technologies because the physical and chemical properties of CECs may be unknown or poorly defined. In other cases, synergies and antagonisms between engineered treatment options and natural attenuation are not understood.

## Identified Future Priorities

The following broad efforts are not discrete tasks that can be usefully prioritized but are interrelated needs, such that addressing one priority assists in addressing the others. We consider only the technical issues associated with avoiding another generation of Superfund sites. We intentionally do not consider the policy, budgetary, and jurisdictional decisions required to implement such priorities.

### Develop CEC priority list and process for list evolution

Foremost, there is a need to expand the scope of hazardous waste site remediation efforts (e.g., the Superfund Research Program) to include CECs that occur at current sites or could potentially create new hazardous waste sites. This will necessarily include developing a list of priority CECs of hazardous waste site relevance. It is imperative that such a list continuously evolves to accommodate new knowledge regarding the occurrence, behavior, and impact of new CECs. It is important to emphasize that models and fixed algorithms alone will not suffice, but serendipity and expert judgment also will play important roles.

The development of a CEC priority list will be a staged evaluation. First, a process is needed for identifying and promoting waste-site–relevant CECs to a priority list. The process by which chemicals are placed on and then, after study, demoted or promoted from a candidate list may be similar to that used by the U.S. EPA for drinking water contaminants or to the Department of Defense for emerging contaminants (Cunniff and Asiello 2009; U.S. EPA 2010b). These approaches ensure continuous reevaluation of the list and suggest a method for selecting candidates from the universe of chemicals (National Research Council 2001). Regular revision of the list is vital to maintain its relevance as knowledge and technology evolve. This general strategy is relevant to both remediation of CECs at existing hazardous waste sites and prevention of new hazardous waste sites caused by the presence of CECs. Abandoning current efforts directed at traditional hazardous waste site contaminants is not advocated; rather, a carefully considered and orchestrated expansion of efforts to identify, evaluate, monitor, and manage CECs of hazard waste site relevance is needed.

### Evaluate potential sources for localization of CEC concentrations

The next generation of hazardous waste sites will not originate primarily from unintentional spills and illicit disposal. Heightened public awareness, greatly improved regulation and oversight, and more environmentally conscious industrial processes have curtailed many practices that led to our current inventory of hazardous waste sites. However, a plethora of chemicals are in commercial use for which little is known, many new processes and disposal practices are coming online that pose unexplored waste discharge problems, and medical end points and modes of action relative to environmental insults are constantly being revised. In addition, new industrial operations, such as e-waste recycling centers, nanomaterial manufacturing, and high-density food production, are largely unevaluated as sources of concentrated CEC contamination. Furthermore, the lack of monitoring for CECs emanating from traditional points of waste accumulation such as landfills and biosolids disposal sites may hide future sources of environmental contamination and exposure.

### Increased and integrated focus on prevention

It is important to acknowledge that the normal means of quantifying success of an increased research effort, such as the number of sites in which new tools enhance the remediation effort, or the local reduction in environmental concentration, will be ill-suited for evaluating success of efforts to prevent future Superfund sites. The most cost-effective and desirable outcome is the prevention of a Superfund contaminant from ever entering the environment, so the relevant (but unmeasurable) metric is the number of sites that do not need to be remediated. Thus, quantification of environmental improvement will be impossible to measure when success is greatest. Ultimately, prevention will depend upon a thorough, holistic understanding of these chemicals through production, use, and disposal. Rigorously reviewed, enhanced life-cycle assessments of CECs considering cradle-to-grave impacts will play a role. Appropriate response and management will manifest as substitution of high-risk chemicals and practices for benign alternatives, revision of commercial and domestic behavior to minimize production and releases of CECs, and an emphasis on reasoned precaution rather than remediation.

## Figures and Tables

**Table 1:** Attributes of CECs of Superfund relevance

High-volume production (surrogate for occurrence quantity) Persistence in a compartment with likelihood of exposure Bioavailability and bioaccumulation Toxicity Localized accumulation with likelihood of exposure

**Table 2:** Research needs identified.

Detection and quantification

Final disposition of CECs
Bioassay-directed methods

Environmental fate and transport

Transformation processes
Fate and transport models

Health effects and risk assessment

Unconventional responses and impacts
Bioaccumulation models

Site investigation and remediation approaches

Epidemiologically and ecologically focused geospatial analysis
Remedial technologies
